# SYMPATHIQUE: image-based tracking of symptoms and monitoring of pathogenesis to decompose quantitative disease resistance in the field

**DOI:** 10.1186/s13007-024-01290-4

**Published:** 2024-11-10

**Authors:** Jonas Anderegg, Radek Zenkl, Norbert Kirchgessner, Andreas Hund, Achim Walter, Bruce A. McDonald

**Affiliations:** 1 Plant Pathology Group, Institute of Integrative Biology, ETH Zurich, Zurich, Switzerland; 2 Crop Science Group, Institute of Agricultural Sciences, ETH Zurich, Zurich, Switzerland

**Keywords:** Disease phenotyping, Precision phenotyping, Partial resistance, Lesion growth rate, Field phenotyping

## Abstract

**Background:**

Quantitative disease resistance (QR) is a complex, dynamic trait that is most reliably quantified in field-grown crops. Traditional disease assessments offer limited potential to disentangle the contributions of different components to overall QR at critical crop developmental stages. Yet, a better functional understanding of QR could greatly support a more targeted, knowledge-based selection for QR and improve predictions of seasonal epidemics. Image-based approaches together with advanced image processing methodologies recently emerged as valuable tools to standardize relevant disease assessments, increase measurement throughput, and describe diseases along multiple dimensions.

**Results:**

We present a simple, affordable, and easy-to-operate imaging set-up and imaging procedure for in-field acquisition of wheat leaf image sequences. The development of Septoria tritici blotch and leaf rusts was monitored over time via robust methods for symptom detection and segmentation, spatial alignment of images, symptom tracking, and leaf- and symptom characterization. The average accuracy of the spatial alignment of images in a time series was approximately 5 pixels (~ 0.15 mm). Leaf-level symptom counts as well as individual symptom property measurements revealed stable patterns over time that were generally in excellent agreement with visual impressions. This provided strong evidence for the robustness of the methodology to variability typically inherent in field data. Contrasting patterns in the number of lesions resulting from separate infection events and lesion expansion dynamics were observed across wheat genotypes. The number of separate infection events and average lesion size contributed to different degrees to overall disease intensity, possibly indicating distinct and complementary mechanisms of QR.

**Conclusions:**

The proposed methodology enables rapid, non-destructive, and reproducible measurement of several key epidemiological parameters under field conditions. Such data can support decomposition and functional understanding of QR as well as the parameterization, fine-tuning, and validation of epidemiological models. Details of pathogenesis can translate into specific symptom phenotypes resolvable using time series of high-resolution RGB images, which may improve biological understanding of plant-pathogen interactions as well as interactions in disease complexes.

**Supplementary Information:**

The online version contains supplementary material available at 10.1186/s13007-024-01290-4.

## Background

Most pathogens undergo multiple cycles of reproduction during the growing season of a crop, and these reproductive cycles fuel seasonal epidemics. Quantitative disease resistance (QR) primarily represents the host’s ability to slow down the progression of these epidemics by limiting the build-up of secondary inoculum responsible for new infections [1–3]. Parameters determining this buildup include infection frequency, latent period duration, initial lesion size, lesion and sporulating area expansion dynamics, spore production in symptomatic tissue, and infectious period duration [1, 3, 4]. Accordingly, QR is a complex trait arising from a combination of factors that influence these parameters during the growing season (‘components’ of resistance [5]).

A decomposition of the overall degree of QR observed at relevant crop developmental stages into these contributing component traits is challenging, particularly under field conditions, because individual leaves and symptoms must be tracked over time for this purpose [6–9]. Yet, such a decomposition can support understanding of the mechanisms underlying a particular form of QR, aiding the identification of distinct and complementary mechanisms of partial resistance for their targeted combined use [5, 10]. In addition, individual components of QR such as lesion expansion rates and the duration of critical periods in the reproductive cycle may be more easily modelled as a function of environmental variables (e.g., temperature and humidity; [6, 8, 11]) than the complex composite trait itself. Explicitly modelling the dependency of the relevant epidemiological factors on key environmental variables could account for much of the often substantial genotype-by-environment interaction in QR (see e.g [12]), thus making the outcome for a particular genotype in a particular environment more predictable.

By default, disease assessments in breeding programs are made at coarse spatial and temporal scales and with a strong focus on aspects of the disease considered most relevant in terms of crop yield and quality at the timepoint of assessment, relying largely on visual scorings [13, 14]. For example, for foliar diseases in wheat, the percentage of the leaf area covered by necrotic lesions or other disorders is typically estimated, implicitly or explicitly combining disease incidence and conditional severity (i.e., severity on symptomatic leaves) [14–17]. Image- and reflectance-based approaches to derive digital proxies for such measures have been proposed, but also operate at the canopy scale [15, 18, 19]. While such assessments provide an estimate of the percentage of the photosynthetically active leaf area that is lost due to a disease, they do not enable a decomposition into damage resulting from symptom number and symptom size (i.e., infection frequency and lesion size and expansion rate). Even if this was attempted, it would be difficult based on a single assessment because originally distinct lesions often rapidly coalesce into larger blotches [9].

Numerous studies have used simulation to investigate how hypothetical changes in different epidemiological parameters affect overall disease progression [4, 20]. Others have employed mechanistic, spatially explicit models to predict disease levels as a function of various parameters driving disease progression at the leaf and canopy level [21–24]. Both types of studies provide useful insights into the sensitivity of the outcome to changes in any of the parameters that drive seasonal epidemics and may therefore help identify the most effective components of QR. Some of these studies also consider interactions in disease complexes, such as in co-infections of wheat leaves with *Zymoseptoria tritici* and *Puccinia triticina* [21]. However, there are few field data available to support the parameterization, fine-tuning, and detailed functional validation of such models. Many values used for model parameterization originate from theoretical considerations or are based on small-scale experiments typically performed under controlled environment conditions with a limited range of host and pathogen genetic diversity [22, 25]. One of the most extensive efforts to collect field-based data on single epidemiological factors was reported by Adhikari et al. [6] who tracked more than 5,000 *Septoria nodorum* blotch lesions on wheat leaves by making more than 18,000 manual measurements of lesion size. Unfortunately, such measurements – although highly insightful - are extremely laborious, may be prone to subjectivity and other sources of experimental error, and are very difficult to reproduce.

Compared to visual scorings and other forms of manual measurement, image-based approaches offer significant potential to standardize disease assessments through objective technical analysis of disease symptoms, increase measurement throughput and precision, and describe multiple aspects of a disease (or even multiple diseases) based on a single measurement in a reproducible manner [26–28]. Previous studies for wheat foliar diseases used analytical image analysis approaches to segment necrotic lesions caused by *Z. tritici* and count fruiting bodies within lesions [16,27,29], detect yellow halos surrounding the lesion perimeter [30], or detect rust pustules caused by *Pucciniales* [31] in images acquired under highly standardized conditions. Because of the high requirements in terms of image standardization, these methods typically do not support repeated assessments of the same leaf since the leaves have to be removed from the plants for imaging. More recently, the adoption of end-to-end trainable deep-learning-based approaches has resulted in significant improvements of these methods, both in terms of accuracy and robustness [31–33]. In particular, such approaches enable accurate and robust symptom recognition even under less controlled conditions such as variable outdoor lighting or in the presence of co-infections with multiple diseases, and can account for substantial variability in symptom and leaf properties [34]. As a result, processing image time series collected directly under field conditions without detaching the leaves of interest is now becoming feasible.

Automatically tracking individual symptoms in a series of leaf images requires all images of the series to be transferred to a common coordinate system in a process called image registration. This step may be avoided if a fixed imaging set-up is used, if lesions are tracked manually, or if re-identifying relatively large symptoms is sufficient, see e.g [35]. In contrast, for large amounts of field-collected data, manual symptom tracking is not feasible and fixed measurement set-ups cannot be used on large sample sizes. More detailed investigations of symptoms or pathogenesis that require a high spatial precision across the entire leaf also demand careful image registration, e.g [36, 37].

Here, we describe (i) a simple, affordable, and easy-to-operate imaging set-up and imaging procedure for in-field acquisition of leaf image sequences that enable tracking of individual disease symptoms, with a throughput of at least 100 images per working hour, and (ii) methods for image registration, symptom tracking, and extraction of biologically relevant leaf and symptom level data from image sequences. As outlined above, we believe that these tools and extracted data can support (i) the decomposition of QR into different component traits, (ii) the quantification of effects of key environmental variables on individual components of epidemiological development, (iii) better understanding of symptom phenotypes and related plant-pathogen interactions, (iv) an examination of interactions in disease complexes, and (v) parameterization and validation of mechanistic models of disease progression.

## Methods

### Plant and pathogen materials

All data was collected in a field experiment carried out during the 2022–2023 wheat growing season at the ETH Research Station for Plant Sciences at Lindau-Eschikon, Switzerland (47.449 N, 8.682E, 520 m a.s.l.; soil type: eutric cambisol). The experiment was a modified repetition of an experiment described in detail earlier [19], augmented with additional cultivars. Briefly, 24 registered wheat cultivars selected for their contrasting morphology and susceptibility to *Z. tritici* were grown in nine replicate plots each. Plots were sown with a drill sowing machine in nine rows per plot with a row length of 1.7 m and a row spacing of 0.125 m at a density of 400 plants m^2^ on 18 October 2022. They received one of the following treatments: (i) a control treatment consisting only of an early fungicide application at jointing (growth stage [GS] 31; [38]), without pathogen inoculation (hereafter called F_0I), (ii) an artificial inoculation with a *Z. tritici* spore suspension before the appearance of the flag leaf (approximately GS 37) without any fungicide applications (0F_I), and (iii) a fungicide application at jointing followed by an artificial inoculation after full flag leaf emergence (GS 39; F_I). The fungicide (Input, Bayer) was applied on 21 April 2023. Inoculations with a mixture of ten different strains of *Z. tritici* were made on 28 April and on 14 May 2023. All leaf rust infections were natural. For further details regarding the experimental design refer to [19].

### Leaf identification, leaf preparation and image acquisition

The main objective of this study was to develop a method for in-field tracking of individual disease symptoms over time to facilitate the investigation of symptom development as affected by various factors including meteorological conditions, leaf number, leaf age, and host genotype. In addition to the image processing methodology itself, an important objective was to obtain an understanding of the required duration and frequency of measurements as well as the number of replicate measurements needed to resolve differences. Therefore, we selected cultivars, plots and leaves with the aim of obtaining a diverse data set encompassing variation in all these factors. We aimed to collect statistically relevant sample sizes for each cultivar.

Image data was acquired over a period of 34 d between 25 May 2023 and 28 June 2023. Images were taken at 31 time points corresponding to 29 different days. On two days, leaves were imaged twice, once in the morning and once in the afternoon, with a time difference of approximately 6 h. Generally, at the start of an image series, we selected intact, fully developed symptom-free leaves in plots with a high incidence of fresh STB symptoms on the leaf layer of interest. We aimed to image 10 leaves within each of three replicate plots for each of the selected cultivars. After selecting the cultivars and plots, single plants were tagged by attaching a numbered red duct tape label to its stem. The corresponding leaf was then prepared for repeated imaging by applying white dots to both edges of the adaxial side of the leaf along a distance of ~ 20 cm, with a spacing of ~ 1.5–2 cm, resulting in ~ 20–30 marks applied to each leaf. These marks were made with a white paint marker with a stroke width of 0.8 mm (Edding, Ahrensburg, Germany), which enabled the addition of a mark without exerting physical pressure on the leaf. Most of these marks remained visible throughout the entire measurement period. Identifying and preparing suitable leaves for imaging took approximately 30 s per leaf.

Measurements were made in batches of leaves according to three different starting dates, as follows (some temporal overlap between batches occurred, but separate batches generally represent different periods of time): For batch 1, penultimate leaves were inspected for the presence of STB symptoms on 25 May 2023, corresponding to late booting and early heading. Selected plots belonged to seven cultivars, which were represented by 2–3 experimental plots, in which approximately 10 leaves were tagged. A total of 177 leaves were analyzed in this first batch. All experimental plots in this batch had received the 0F_I treatment (i.e., no fungicide followed by an early inoculation). Measurements were made almost daily until 8 June 2023. A final measurement was made on 13 June 2023. On average, 15 images were acquired for each leaf. For batch 2, 179 flag leaves belonging to 6 cultivars and 3 replicate plots for each cultivar were selected on 8 June 2023, which corresponded to very early grain filling. An average of 15 measurements were made for leaves contained in this batch during the time that lasted to 28 June 2023. Finally, for batch 3, 30 flag leaves belonging to one cultivar and three replicate plots were selected on 20 June 2023 which corresponded to mid grain filling. This batch was limited to a single cultivar because of the very high incidence and severity of brown rust infections and the onset of physiological senescence in all other cultivars. The last measurement for this batch was made on 28 June 2023. In total, 5458 images were available.

Images were acquired using a simple custom-developed set-up similar to a set-up described previously [34]. It consisted of a 3D-printed spacer that could be mounted directly onto the camera lens and a compatible acrylic glass base plate (Supplementary Figure S1). The base plate served as a background against which the leaf could be flattened. Flattening of the leaves was achieved by pressing the leaf gently against poster gums placed on the plate. The base plate had three wells into which the spacer could be inserted for stabilizing the camera, which allowed images to be taken at a constant working distance and with constant orientation. This set-up was easily operated by a single person and enabled the imaging of approximately 100 leaves per hour. Images were taken using a full-frame mirrorless digital camera (EOS R5, Canon Inc., Tokyo, Japan; 45 megapixels, 36- × 24-mm sensor) combined with a macro lens (RF 35 mm f/1.8 IS Macro STM, Canon Inc., Tokyo, Japan). This setup resulted in a physical resolution of approximately 0.03 mm/pixel. On the first two measurement dates, exposure times were around 1/80 s. This sometimes resulted in blurred images which became apparent only upon closer inspection of images after completion of the measurements. Therefore, exposure time was subsequently fixed to 1/160 s. The F-stop was fixed to 8 or 7.1, which allowed keeping ISO speed below 1000 in most cases, preventing significant levels of background noise in captured images.

### Image registration

All images in a time series were co-registered to the first image of the series in two steps: (i) detection of the artificial white marks on the leaves and recording of their image coordinates, and (ii) the actual registration of the images using the detected marks as matching pairs of points to estimate a transformation. Though implementation details differ to account for natural conditions and variation in leaf properties, our procedure for image registration is similar to the one proposed by Behmann et al. [36] for spatial referencing of hyperspectral image series collected under controlled environment conditions. The quality of the image registration was quantitatively assessed by manually tracking 225 prominent features (e.g., pycnidia, prominent leaf hairs or an edge of a damaged zone) in 55 randomly selected pairs of images that originated from different timeseries. Image coordinates of these features were recorded using a simple custom-developed tool based on matplotlib v3.5.0. Euclidean distances between the respective positions retrieved from each of the two images were measured and translated into physical distances assuming a physical resolution of 0.03 mm/px.

For the detection of the white marks, a YOLOv8s Pose model [39] was trained. All visible marks in 133 selected example images were annotated as points by clicking once at the estimated centroid of the mark, using the computer vision annotation tool (CVAT, https://www.cvat.ai/). Annotations were exported in ‘CVAT for images 1.1’ format and exported to YOLO-Pose dataset format using a custom-developed python script. Preparing, annotating, and post-processing the annotations took approximately one day. The model was then trained using 115 randomly selected images (~ 86%) for training, with default settings and batch size 3 for 184 epochs, at which point no improvement in performance had been observed for 30 epochs of training. The only adjusted parameter was image size, which was optimized to 2016 px, thus reducing the original resolution by a factor of 4. The model reached a validation F1-Score of 0.98 on the 18 validation images. During prediction of the full data set, a lower-than-optimal confidence threshold (0.3 instead of 0.43) was used in favor of a higher recall at still acceptable precision.

False positive marks lying outside of the leaf were identified and removed from the list of detected marks by comparing their x- and y-coordinates with the median of all detected marks. Maintained detected marks delimited the region of interest (ROI) on the leaf that was to be used for further analyses. In each image, the minimum area bounding rectangle containing all detected marks was fitted. The image and the bounding rectangle were then rotated about the center of the image for the bounding box to have an angle of 0°. The rotation matrix and the adjusted bounding box coordinates were saved in a JSON file. Image coordinates of the detected marks were transformed to coordinates for the rotated ROI by applying the same rotation matrix followed by a translation in x- and y- direction based on bounding box image coordinates. This ended the process for the first image in each series. All subsequent images in a series were co-registered to this target ROI by searching for matches between detected marks, using KD-Trees [40]. If all but a maximum of two detected marks could not be matched to the reference image and the ROIs had similar dimensions, then the point pairs were directly used to estimate (i) a projective transformation, (ii) a polynomial transformation, and (iii) a piecewise affine transformation. The estimated parameters were saved for later re-use. If insufficient pairwise matches were found and/or the ROIs had different dimensions, images could not be directly matched to the first image of the series. Instead, an attempt was made to first register the image with the preceding image in the series. This was achieved by detecting scale invariant feature transform (SIFT) features [41] in both images. Matching SIFT features across the images were then processed using the random sample consensus (RANSAC) algorithm [42] to find the homography matrix between the two images. Like before, the coordinates of the detected marks were transformed using the estimated homography. The search for matches between marks was then repeated, and the image co-registered to the target image, as described above.

### Symptom detection, segmentation, and tracking

All original, untransformed images captured with the described set-up were cropped to patches sized 8192 × 2048 pixels that contained the entire imaged leaf area, using the exported ROI bounding box coordinates as a guide for cropping. These patches were then processed using deep learning based semantic segmentation and key point detection models trained on the same type of leaf images in previous work [34] for segmentation of the leaf, necrotic areas, and insect damage, and for the detection of pycnidia and rust pustules, respectively. The resulting predictions were visually inspected and obvious failure cases were identified and addressed in an iterative process. In the first iteration, 48 patches sized 1224 × 1224 pixels with typical but mostly very large symptoms were annotated and added to the data set described elsewhere [34]. In a second iteration, another 32 patches sized 1024 × 1024 pixels were annotated, with the aim of (i) reducing the false positive rate in cases of insect damage and other physical damage to the leaves that did not represent necrosis, and (ii) improving the performance on leaves showing prominent signs of physiological senescence. The added training samples represented cases that were rare or absent in the original training data set, which was sampled from a single season. The models were then retrained following the procedure described by Zenkl et al. [34]. The resulting masks and the 2D-coordinates of detected points (pycnidia and rust pustules) for each series of images were aligned by applying cropping, rotation and transformation as determined during image registration (see above).

Tracking individual symptoms in co-registered subsequent images of the same leaf faced two challenges: First, image registration was imperfect, resulting in small shifts of a given physical position on the leaf between subsequent images. This problem was more pronounced when global projective and polynomial transformation was used for image registration, since this type of transformation cannot account for local deformations. Second, small, initially separate lesions typically grew towards each other and coalesced over time to form single, large blotches. In extreme cases, an initially separate lesion became completely engulfed by other lesions growing around it. Both issues were addressed by comparing each mask with the preceding mask of the same leaf. In the first case, if for any given lesion in the current mask, a lesion in the preceding mask could be found with an area overlap with the current lesion of more than 30%, it was considered to represent the same lesion and was assigned the same label. The second issue was solved by performing watershed segmentation of the current mask, using the preceding segmentation mask as the starting point from which to flood basins. Watershed segments were separated by a three pixels wide line to maintain separation between coalesced lesions. Visual inspections of several image series showed that this simple post-processing was highly effective and enabled us to correctly track individual lesions over time in nearly all cases. Very small predicted necrotic areas measuring less than 1000 pixels were removed, as these very often represented false positives that were not detected in subsequent images of the series. However, correctly identified small lesions were also ignored as a result. This threshold value thus balances the sensitivity and robustness of the analysis.

### Lesion and lesion edge characterization

After identifying a lesion as representing an already tracked or a new object in the image series, leaf and lesion characteristics were extracted and exported for further analyses. At the leaf level, measures relating to the areas affected by different types of damage and the total number of different symptoms as well as density and distance metrics between individual symptoms were exported. At the lesion level, we extracted the contour area, the contour perimeter length, the solidity, height and width, and the number of contained pycnidia. Given the interest in the dynamics of symptom development, additional information characterizing each lesion’s spatial context was extracted. For example, knowledge on the immediate spatial context of each lesion is required for a correct interpretation of lesion expansion. Specifically, lesions cannot grow freely and uniformly in all directions if they are located on the leaf edge or on the edge of the image, if there is insect damage or other lesions nearby, or if the lesion has a complex shape. To identify such regions of the lesion perimeter, we adapted methods described in detail earlier [30]. Briefly, the lesion contours were approximated by fitting b-splines to the contour pixel coordinates. Then, the leaf mask and the Euclidian distance transform of the inverted segmentation mask were sampled on the spline normals in outward direction, to a distance of 15 pixels from the lesion perimeter. This identified portions of the perimeter that could not sustain uniform radial growth of the lesion. The fractions of the total lesion perimeter affected by these limitations were exported as an additional lesion characteristic. Here, rust pustules were not considered to represent a barrier for STB lesion expansion, following the rationale outlined by Garin et al. [21].

### Implementation details, code, and data availability

All image processing and analysis was performed using OpenCV v4.8.0.74 [43], scikit-image v0.20.0 [44], and scipy v1.11.1 [45]. Formal analyses and visualizations were performed using R v4.1.2 [46]. Leaf and symptom segmentation and detection was performed using the LeafToolkit [34]. All code related to the processing of image time series and leaf- and lesion-level trait extraction is available from https://github.com/and-jonas/sympathique-wheat for documentation. A sample data set and the trained reference mark detection model can be downloaded from ETH research collection at 10.3929/ethz-b-000659812.

## Results

### Image registration

**Fig. 1:**
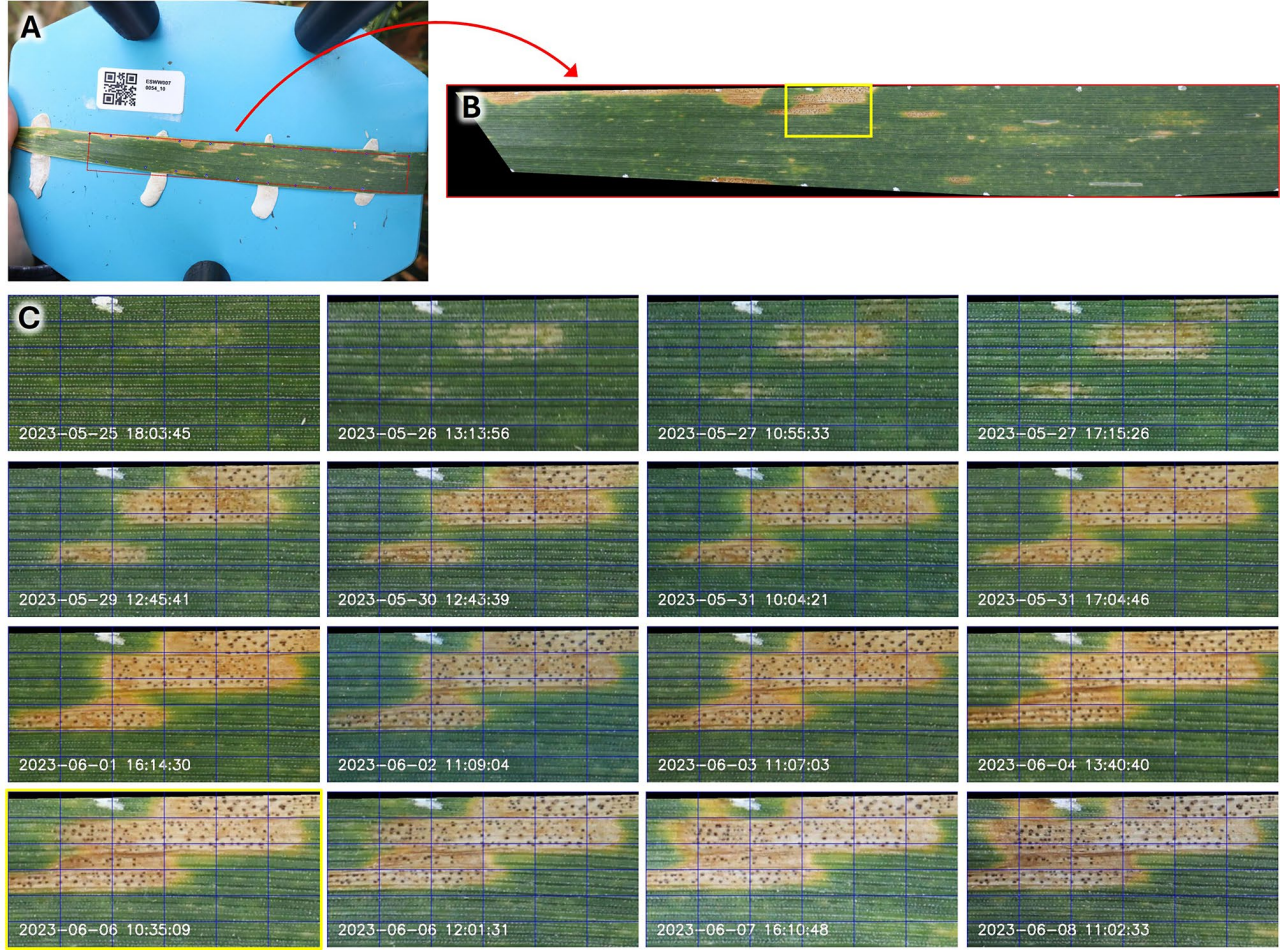
Example result of the implemented workflow for image registration. **(A)** An original image with detected artificial marks highlighted by blue circles and the fitted minimum area bounding rectangle delimiting the region of interest highlighted in red; **(B)** One image of the series that has been co-registered to the first image of the series by cropping, rotation, and a piece-wise affine transformation, using the detected marks as shown in **(A)**. Black parts of the image denote regions outside of the convex hull defined by the detected marks that cannot be analyzed when using piece-wise affine transformation for image registration; **(C)** a constant region of the leaf exhibiting developing *Z. tritici* lesions with pycnidia, highlighted in yellow in **(B)**. The blue grid was added to facilitate visual estimation of the quality of image registration and examination of changes between subsequent images in this series. Date-times indicated in each panel indicate the time point of image acquisition

The proposed approach for symptom tracking (cf. Figure 1A and B) relies on area overlap of detected objects across subsequent images for their correct identification and labelling.

Accordingly, a high precision of image registration across the entire image is critical for the performance of the method, especially considering the sometimes-microscopic scale of symptoms. Local and global (i.e., whole leaf) deformations, especially at advanced stages of leaf or symptom development led to large errors when global transformations were used for image registration. As a result, it was not possible to define a globally meaningful threshold for the minimum area overlap required for an object in a series to be correctly identified as an already tracked object. Therefore, global transformations were abandoned without detailed quantitative evaluation. Instead, piecewise affine transformations were used, thus making the best use of the spatially dense pattern of markers applied to the leaf to account for both global and local deformations.

**Fig. 2:**
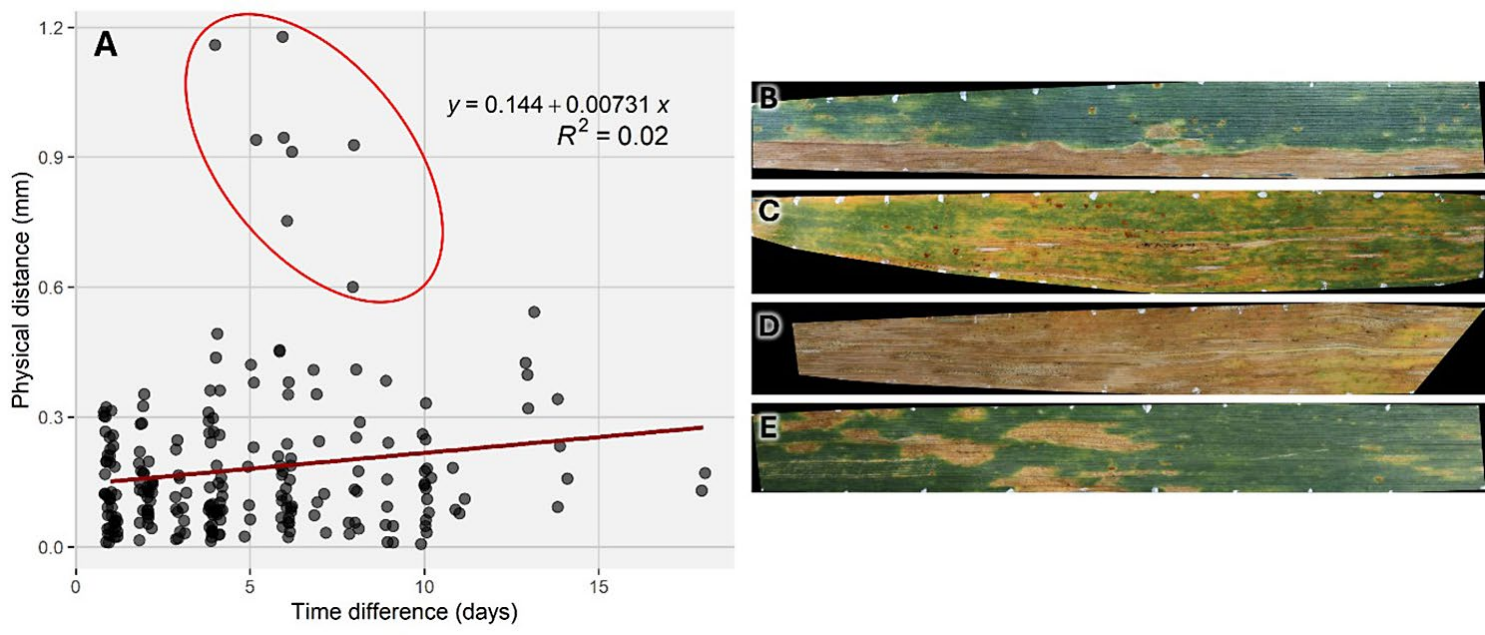
Evaluation of the accuracy of image registration based on manual tracking of prominent features in randomly selected image sequences. **(A)** Physical distance between the positions of 225 manually tracked features retrieved from 55 pairs of images plotted against the time span lying between the two images. The dark red line represents the linear least squares regression of the distance on the time span, with the corresponding equation and R^2^ metric reported on the upper right. Jittering has been applied to data points for better readability. (**B-E**) Images of the leaves from which the data points marked by the red ellipse in (**A**) were retrieved. The image corresponding to the second, later time point for each pair is shown. Note that images (**B**-**D**) represent challenging cases for the detection of the artificial marks; failure to detect a mark results in larger segments for which a uniform affine transformation is estimated. The missing parts at both ends of the leaf in (**D**) illustrate the consequence of undetected marks at the horizontal edges of the region of interest: these parts of the leaf cannot be co-registered to the target

**Fig. 3:**
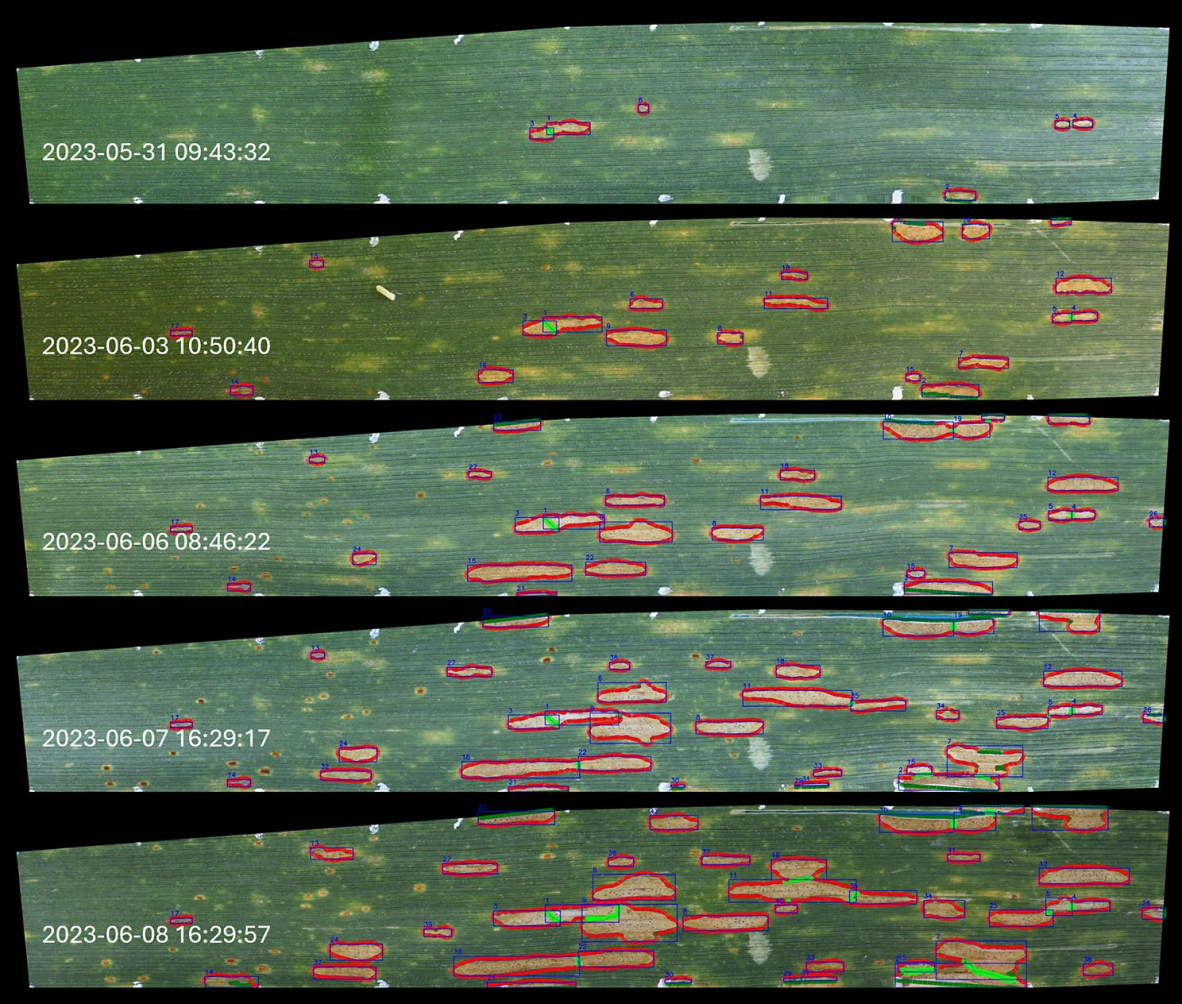
Example result of the implemented *Z. tritici* lesion tracking algorithm on registered images, graphically represented on a subset of available images for one leaf. Smoothed contours of the detected lesions are represented by thin black lines. Short colored lines perpendicular to the smoothed lesion edges represent spline normals with a length of 15 pixels. Red normals represent directions that can support radial growth of the lesions, dark green normals extend outside of the region of interest or outside of the leaf, into insect damage, or into the lesion itself (invaginations), bright green normals extend into neighboring lesions. Lesions are attributed an identifier (blue number on the top left corner of the bounding box for each detected lesion) according to their order of appearance, and identical lesions in subsequent frames keep their identifier if the tracing is successful. Separation between coalescing lesions is maintained through watershed segmentation with the previous mask as a starting point. Note that detected rust pustules and pycnidia are not highlighted here for the sake of clarity. Also, note that inference was not performed on the images displayed here, but on the original untransformed images as shown in Fig. 1A

Manual feature tracking in image pairs allowed to estimate the average error associated with this method to approximately 0.15 mm (~ 5 pixels) and below 0.6 mm (~ 20 pixels) in all but a few cases, with a very weak trend towards a higher error with increasing time span between images (Fig. 2A). Some outliers with errors > 0.6 mm were observed when substantial parts of leaves became symptomatic in a spatially heterogeneous manner (Fig. 2B), with advancing natural senescence and generally widespread disease (Fig. 2C and D), or occasionally when large symptoms led to pronounced local deformations (Fig. 2E). In most cases, the accuracy of image registration was, however, sufficient to allow for detailed examination of symptom development from when first signs appeared to when symptoms started to coalesce (Fig. 1C). Visual inspection of the lesion tracking results suggested a very stable performance and reliable identification and labelling of lesions, even if they were very small at the time of their initial detection (Fig. 3, numbered blue bounding boxes). The principal remaining weakness of the proposed method is that occasional failures to locate individual reference marks at each end of the ROI (horizontally) led to failure to align the corresponding image segment, reducing momentarily the analyzable leaf area. This was the case if the relevant marks were either lost during the measurement period or occluded or simply not present in the image due to operating errors (e.g., imprecise placement of the leaf on the plate, occlusion by a finger, etc.), see Fig. 2D. Similarly, occasional false positives located close to true positive marks that could not be filtered out based on their 2D coordinates lead to deformations within single segments of the leaf. In contrast, failure to associate correctly detected reference marks were rare. Overall, most of the ROI could be properly registered to the reference image in all but a small number of image sequences (Supplementary Figure S2).

### Symptom Tracking

Overall, our pilot data collected to support method development already enabled the monitoring of 13,538 individual necrotic lesions, which provided 72,005 lesion property measurements. Each lesion was measured 5.3 ± 3.8 times (mean ± standard deviation) over a period of 4 d 18 h ± 4 d 8 h. Mean interval duration between two consecutive measurements was 1d 2 h ± 6 h, based on 11,419 lesions (~ 86% of all lesions) that were measured more than once.

Though a detailed phenotypic analysis of symptom development directly on the registered image sequences will likely be very insightful, here we focus our analysis on the extracted leaf and lesion-level traits. This analysis provides a thorough technical validation of our method because image-based patterns over time can be compared with expected patterns to examine the stability and robustness of trait extraction. A pixel-based evaluation was not performed, because this would require a representative sample of image time series to be manually annotated with detailed semantic labels.

**Fig. 4:**
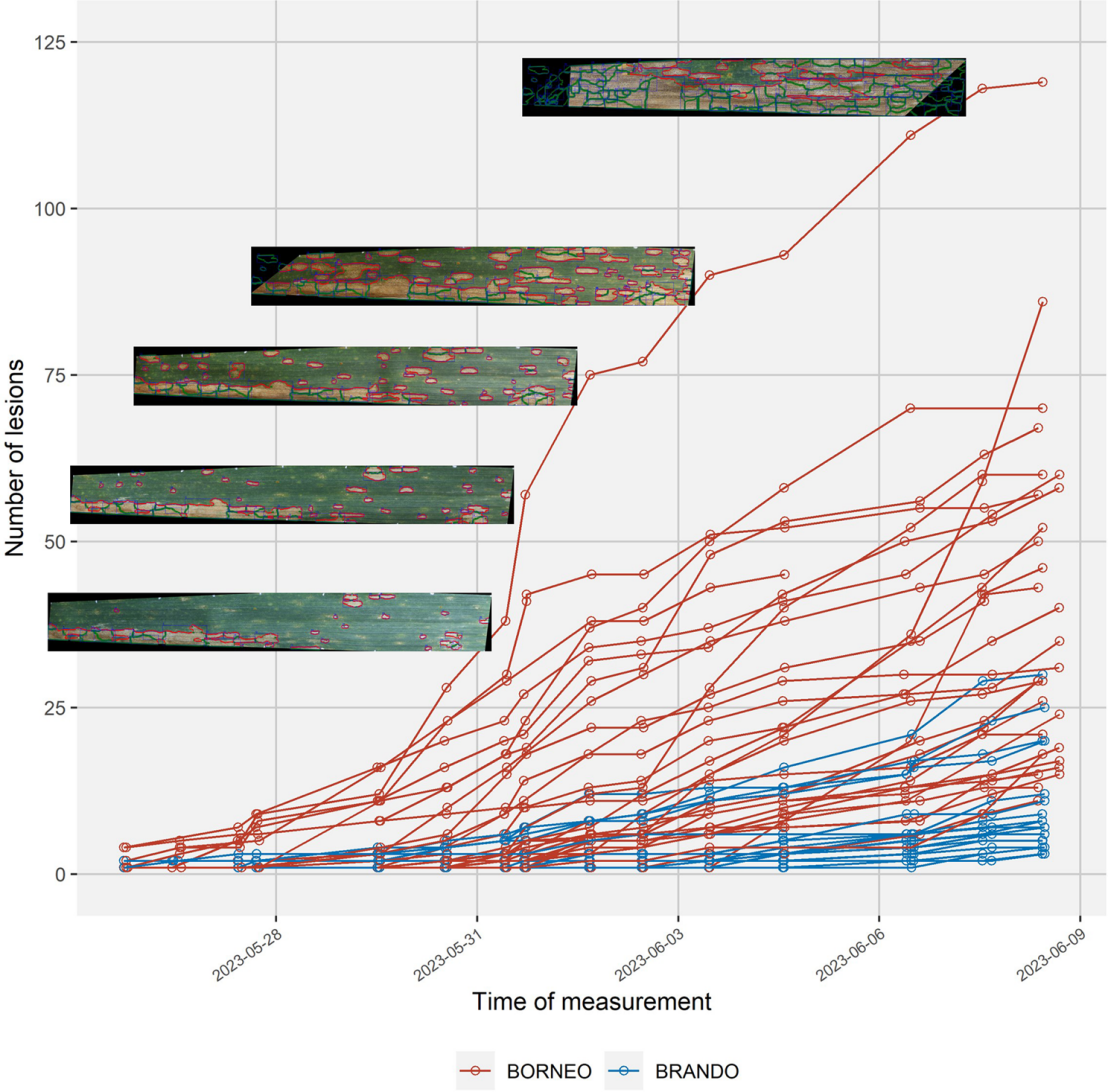
Total number of lesions identifiable in the time series as separate infection events, plotted against the time point of measurement. Each time course represents data obtained from one leaf. Colors represent two different genotypes (‘Borneo’ – susceptible; and ‘Brando’ - resistant). Images show a leaf of the cultivar ‘Borneo’ in correspondence with the most near-by data points. Leaves for each genotype were sampled from three different experimental plots

**Fig. 5:**
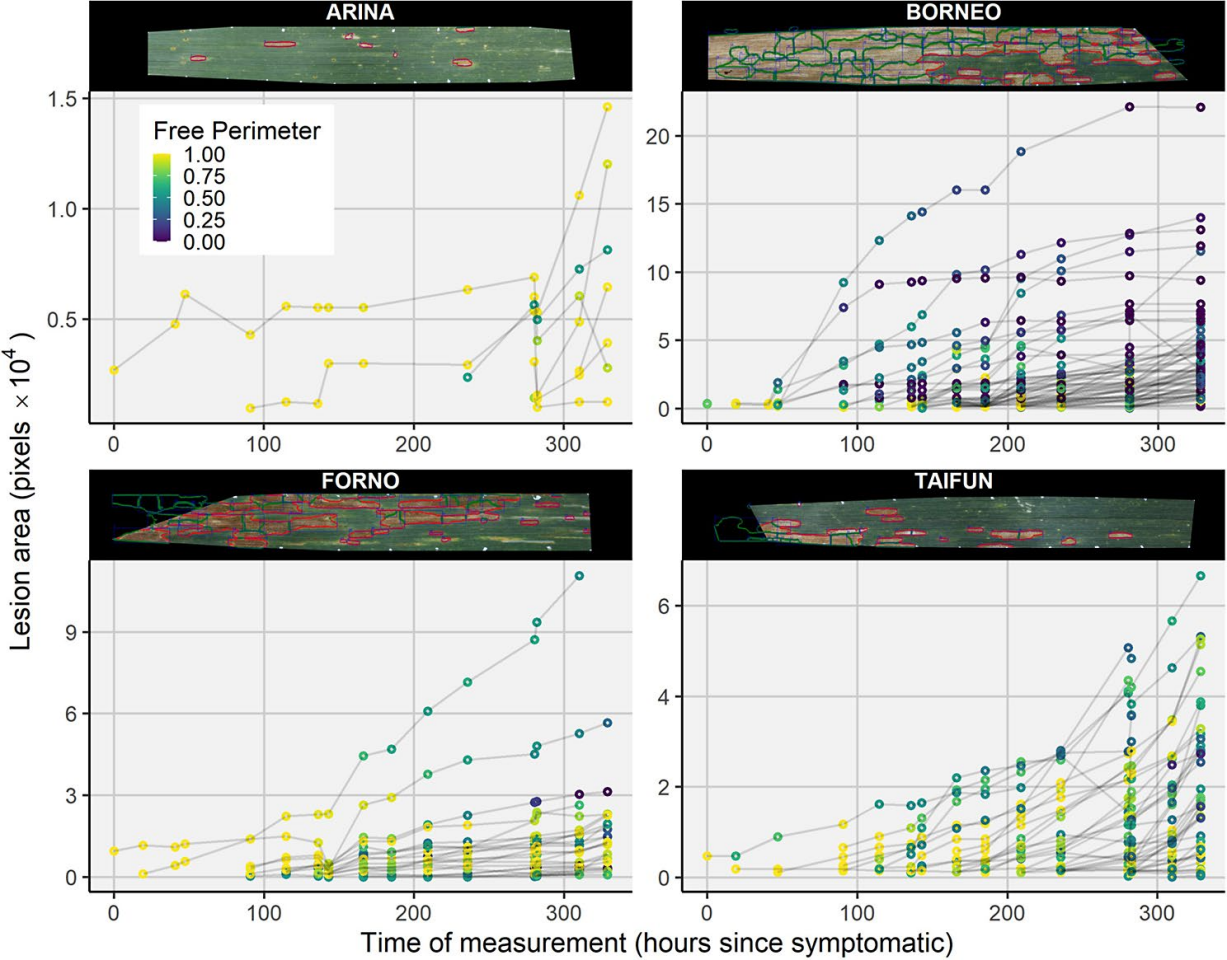
Area of individual lesions plotted against time after first symptoms appeared on each of the leaves. Panels show data for four randomly selected plots from the first sample batch. For each plot, one leaf was randomly selected, of which the last image of the series is included for reference. Note that parts of the images were not successfully aligned due to the loss of the corresponding artificial reference mark over time. Failure to align parts of the image results in missing data (see e.g., for the largest lesion in the bottom right panel). The color of each data point represents the portion of the lesion perimeter that can support further lesion growth. Note that the scale of the y-axis differs among panels

Across all samples, the number of lesions per leaf showed the expected monotonic increase over time in most cases (Supplementary Figure S3). On some leaves, more than 100 individual lesions attributable to separate infection events were detected (Fig. 4, Supplementary Figure S3). These high numbers were in excellent agreement with visual observations in those image series (Fig. 4). More detailed analyses of individual symptoms revealed monotonic lesion growth or stable lesion size over time in most cases (Fig. 5). Rare deviations from this expected pattern included mostly very small lesions, where minor inconsistencies in lesion segmentation across subsequent images of a series sometimes resulted in an unexpected relative shrinkage of lesions (e.g., very small lesions shown in Fig. 5, top left panel). In general, results confirmed visual impressions (cf. Figure 3) that lesion tracking and labelling over time was successful in most cases. Furthermore, they highlighted the robustness of the underlying segmentation and detection models, and further confirmed that the quality of image registration was sufficient for re-identification of lesions in subsequent images based on area overlap.

**Fig. 6:**
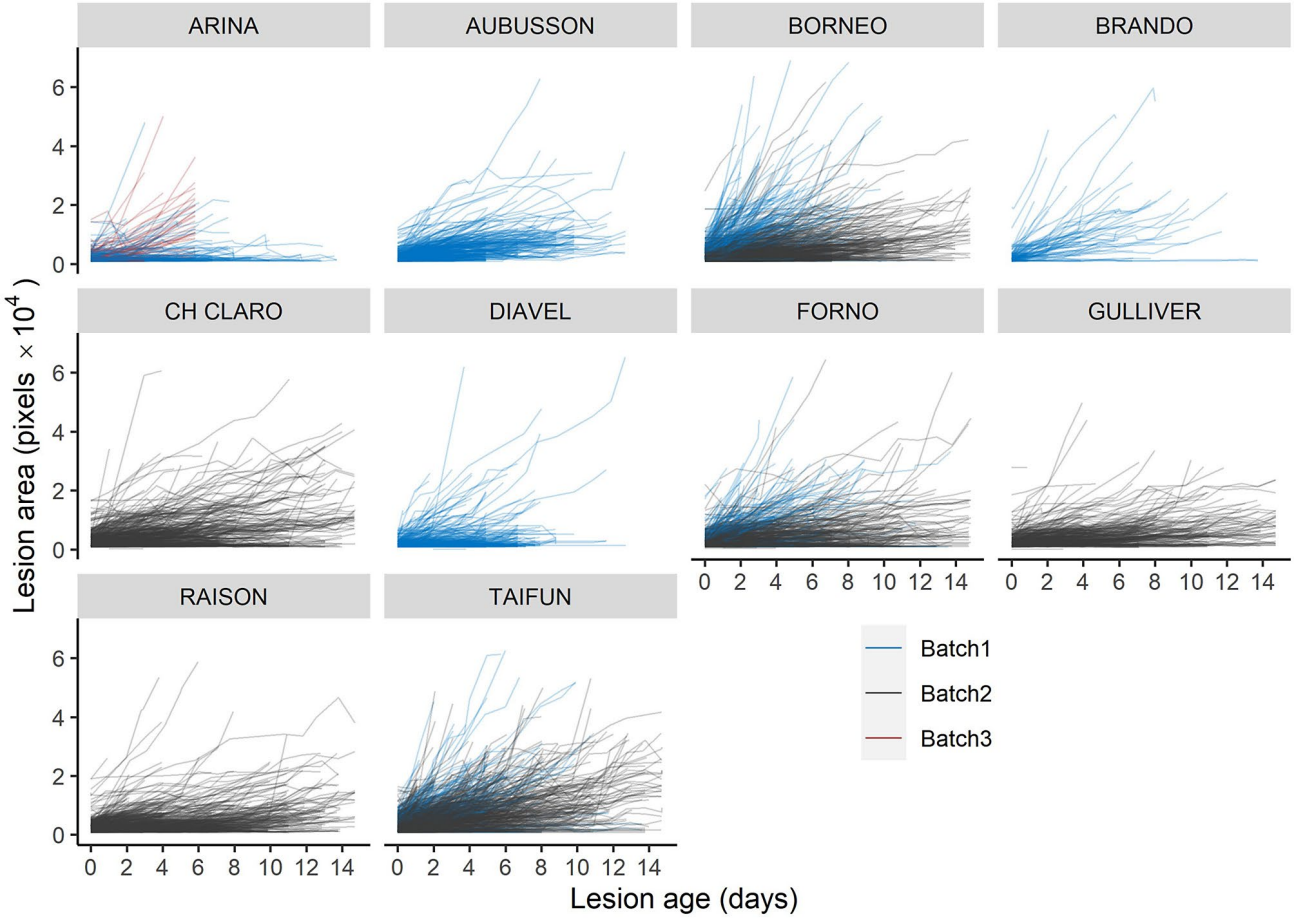
Lesion area as a function of lesion age on ten wheat cultivars. Each line represents a single lesion traced over time. A trajectory for a particular lesion starts when it was first detected, its initial value thus represents the initial lesion size. Note that only data for lesions that have a perimeter which is essentially free of barriers to expansion is shown

Lesion size at any given lesion age showed massive variation, even when single leaves or multiple leaves from a single experimental plot were analyzed (not shown). Large variation persisted even when the analysis was restricted to lesions that had a large portion (> 85%) of their perimeter free of visually apparent barriers to lesion expansion (Fig. 6). Still, some systematic patterns became apparent: First, lesion size as a function of lesion age appeared to depend significantly on the batch, with lesions in batch1 (mostly penultimate leaves around heading) expanding faster than lesions in batch2 (mostly flag leaves, early grain-filling). In batch3 (flag leaves, mid- to late grain filling) lesions tended to expand faster again, though there was relatively little data and only a single genotype available to support this observation. Second, some differences between genotypes were obvious: For example, although many lesions developed on leaves of ‘Aubusson’, their growth was on average much slower than on leaves of ‘Borneo’ (Fig. 6). In contrast, lesion growth was relatively rapid on leaves of ‘Brando’, even though few lesions were able to develop (Fig. 4). It therefore appears that these two genotypes slow down disease progression on leaves in different ways, and this difference can be discovered and accurately quantified using repeated imaging of leaves.

Lesions typically continued to expand over extended periods of time and sometimes for the entire duration of the measurement (Figs. 1C, 5, 6 and 8B), though growth often exhibited irregular patterns over time. Sometimes, lesions grew slowly and steadily over longer periods of time (Fig. 1C). In other cases, lesions expanded very rapidly within very short periods of time (see Supplementary Figure S4 for an example). In such cases, a high measurement frequency is required to correctly identify individual lesions before they coalesce with other lesions to form larger blotches. Overall, our results indicate that lesion growth under field conditions is determined by a multitude of factors in a complex manner. An indication of this is given by the fact that when pooling all available measurement intervals for all tracked lesions, variation in lesion growth during measurement intervals was almost unexplained by the duration of the interval. This was the case even when the observed growth within an interval was corrected to account for the variable portion of the perimeter that was free of barriers to lesion expansion (R^2^ < 0.01, p < < 0.001; Supplementary Figure S5). A similar picture arose when data were analyzed separately for each batch and genotype (not shown).

**Fig. 7:**
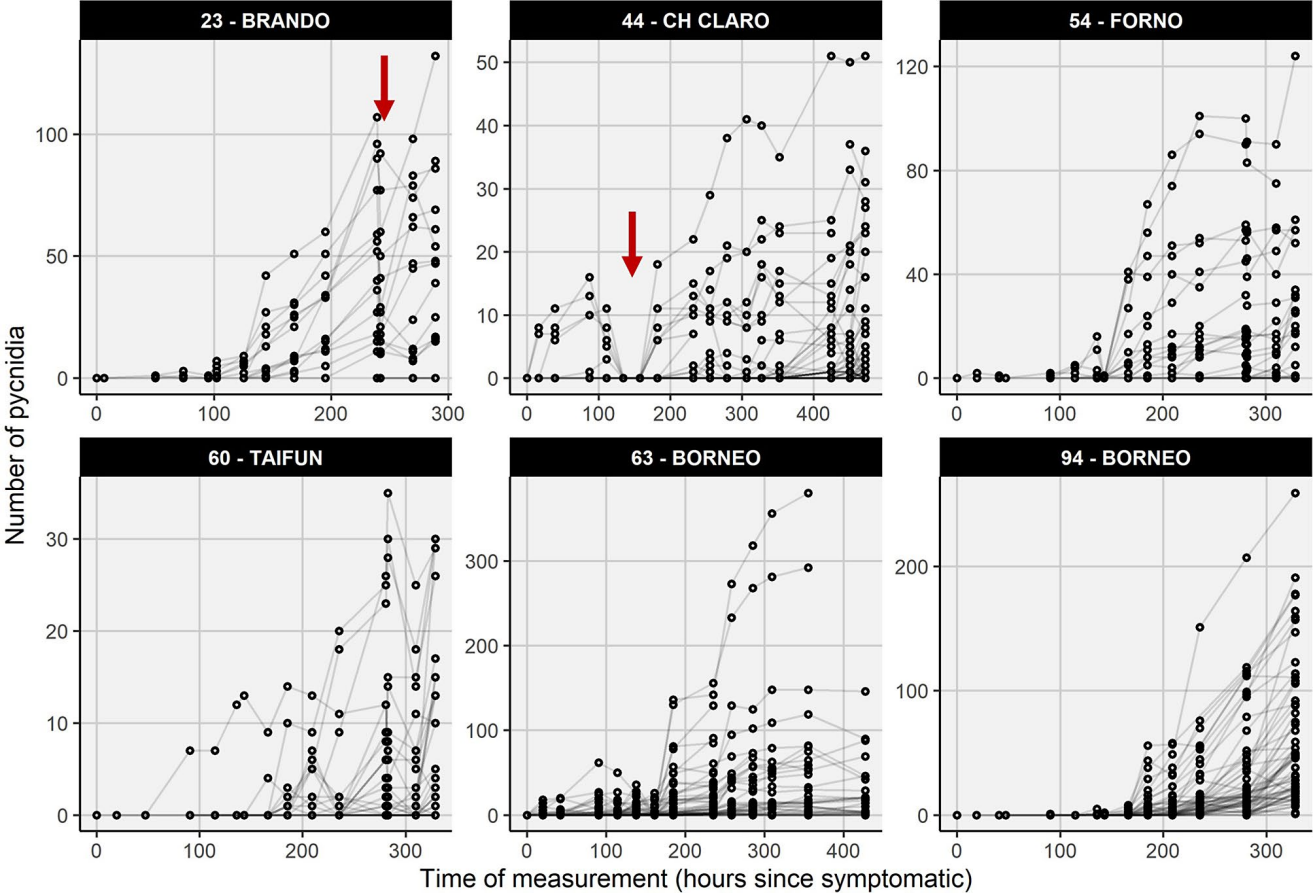
Number of pycnidia within each lesion as a function of time after first symptoms appeared on the leaf, for all lesions in one leaf per plot. Where meaningful (substantial number of pycnidia present) the results for the same leaves as in Fig. 5 are displayed. Strip titles indicate the plot number and the cultivar name. Red arrows mark prominent deviations from the expected monotonic increase in the number of pycnidia per lesion despite a substantial number of pycnidia present. See Fig. 8 and Supplementary Figure S5 for details on the two marked failure cases. Note the scale of the y-axis varies across panels

**Fig. 8:**
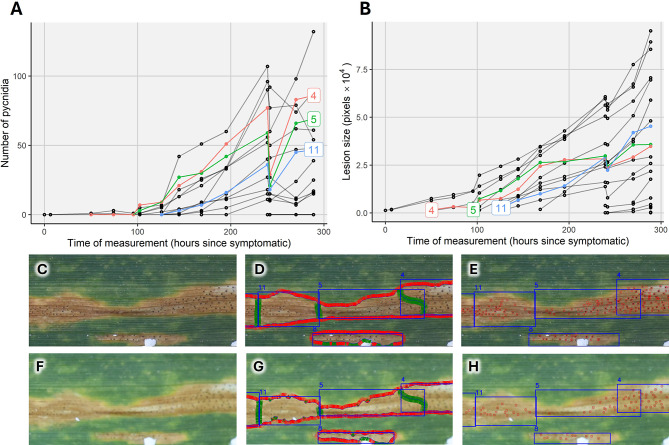
Deviations from expected monotonic temporal patterns in the number of pycnidia per lesion are often related to image quality issues. Here, an operator error resulted in a part of the image being out of focus, which affected pycnidia detection and - to a lesser extent - lesion segmentation and size estimation due to resulting difficulties in image registration; **(A)** Number of pycnidia within each lesion as a function of time after first symptoms appeared on the leaf, for all lesions in the image of interest. The highlighted lesions 4, 5, and 11 (labelled according to their sequence of appearance) showed particularly strong deviations in their pycnidia counts; they are shown in the image cutouts below; **(B)** Lesion size over time, with the same lesions highlighted; **(C-E)** Cutouts from the first image of the two images in question, showing the aligned image and the predictions from the segmentation and point detection models. The three highlighted lesions (numbered blue bounding boxes), with inferred lesion boundaries correspond to the highlighted lesions in (**A**) and (**B**); **(F-H)** Cutouts from the second image taken ~ 3 h later

The number of pycnidia detected within each lesion also showed a stable, monotonically increasing pattern over time in most cases (Fig. 7). Exceptions from this pattern were observed occasionally and could most often be traced back to operator errors in image acquisition (Fig. 8, Supplementary Figure S6). In particular, minor problems with focus settings strongly affected pycnidia detection, which can be well explained by their small size (Fig. 8C-H). Besides these issues, the stable temporal patterns indicate sufficient precision in image registration and processing to allow for monitoring pycnidia number in specific areas of the leaf.

Across the entire data set, a slight trend towards an increased pycnidia density with increasing lesion age was observed, but this trend was inverted in older lesions. As was the case for lesion size, massive variation in pycnidia density for lesions of any age was observed across the entire range of lesion ages (Supplementary Figure S7).

### Leaf-level patterns of disease development

Leaf-level metrics require a constant reference for comparability, and hence require the entire ROI to be correctly registered to the reference image. This was not always achieved using the proposed marking system and marker detection and alignment approach (Supplementary Figure S2), which highlights the need for further improvements of the method in that sense. Here, to ensure a constant reference for leaf-level metrics, we limited our analysis to data from leaves for which at least 90% of the area could be registered to the reference image.

**Fig. 9:**
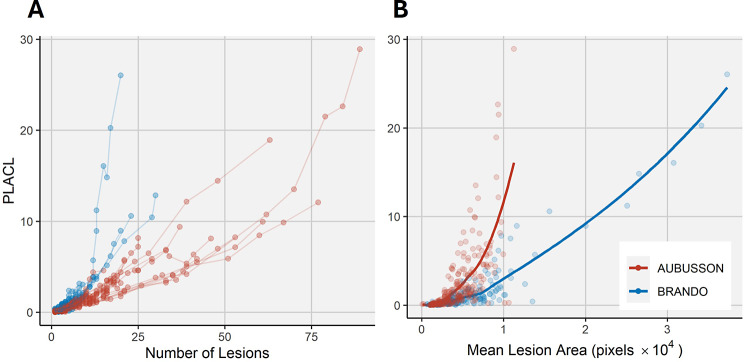
Contributing factors to the percentage leaf area covered by lesions (PLACL) for two contrasting genotypes. **(A)** Relationship between the number of lesions observed within the region of interest and PLACL. Connecting lines trace single leaves over time. **(B)** Relationship between the mean lesion area on a leaf and its PLACL. Loess fits have been added to show the trend of the relationship. Data points in **(B)** have not been connected because average lesion area on a leaf can follow irregular patterns over time as new, smaller lesions appear. Note that, although such analyses would be possible using single time point measurements, repeated measurements are needed to accurately estimate the number of separate lesions that can be traced back to individual infection events

In accordance with the number of lesions and individual lesion size, leaf-integrated metrics of necrosis such as the percentage of leaf area covered by necrotic lesions (PLACL) also showed a monotonic increase over time in almost all leaves (Supplementary Figure S8). Across different cultivars, the number and average size of lesions contributed to total PLACL to varying degrees (Fig. 9), confirming patterns observed at the level of individual lesions (Fig. 6). In particular, though many lesions were detected over time on leaves of ‘Aubusson’, PLACL was on average lower than on leaves of cultivar ‘Brando’ which had fewer lesions on average (Fig. 9A). Conversely, at any given average lesion size, PLACL was substantially higher in ‘Aubusson’ than in ‘Brando’.

The number of rust pustules steadily increased in most leaves, with rapid increases observed later in the season (i.e., for leaves in batch2; Supplementary Figure S9). Slight decreases in the number of rust pustules on some leaves toward the end of the measurement period for each leaf indicated that rust pustule detection in senescing or heavily diseased leaves was more challenging than in younger or less damaged leaves (Supplementary Figure S10). Nonetheless, the rapid development of rust diseases during the later season was well documented.

## Discussion

### Monitoring foliar disease development using time-resolved in-field RGB imagery

The primary aim of this study was to devise a robust and labor-efficient methodology to investigate isolated epidemiological factors under field conditions to decompose QR. Traditionally, similar data have been gathered through visual estimates or manual counts or measurements, for example of lesion or fruiting body number and size [3, 6, 8, 9, 11]. Some form of repeated imaging has been used by a few studies under controlled conditions [35, 47]. Objectivity, reproducibility, and throughput are critical in such assessments because they must be made repeatedly with a high frequency to capture relevant dynamics. An image-based approach holds the potential to significantly increase throughput, making these assessments more feasible for integration into genetic studies or breeding programs [6]. At least in the case of wheat foliar diseases, many of the relevant factors including the duration of critical periods in the reproductive cycle can be readily assessed by repeated visual scoring, indicating that they are in principle amenable to assessment using regular RGB imagery [25, 29]. This holds true particularly for assessments based on the number and spatial extent of lesions. In addition, the number of visible fruiting bodies in combination with their size, color, and spatial arrangement may enable an accurate estimation of spore production in symptomatic tissues over time [25, 29, 48].

A key benefit of RGB-based approaches is that very high-resolution images can be captured in a snapshot manner. Together with the increased availability of suitable image processing algorithms, this greatly reduces the need for standardization of the imaging process. RGB-based approaches can therefore be used directly in the field. This is key here because QR can be significantly affected by canopy-level traits (in addition to leaf- or plant-level traits) that are only fully expressed in field plots [8, 22], and because the dependency of epidemiological factors on environmental variables, like many other biological processes, is arguably best assessed under realistic environmental co-variate courses (as opposed to comparably simple, pre-scheduled, repetitive regimes) [8, 49, 50]. Here, we demonstrate the feasibility of this approach across morphologically contrasting wheat cultivars, changing ambient lighting conditions, phenological growth stages, and with and without inoculation with a diverse sample of pathogen strains as well as co-infections with multiple pathogens. While the proposed approach is transferrable to other crops, it is important to keep in mind that the availability of accurate and robust methods for symptom detection and segmentation represents the backbone of disease development monitoring. Their availability is currently restricted due to the scarcity of meticulously annotated comprehensive training data. As of now, the diseases addressed in this study remain an exception in this regard.

The opportunities for in-field assessments outlined above are somewhat in contrast to the realities of hyperspectral imaging based approaches which require highly controlled conditions for image acquisition [51], but have also been advocated for the study of plant-pathogen interactions [36, 50–52]. We recognize that such approaches are likely to be more suitable for detailed investigations of molecular and physiological processes involved in pathogenesis as well as for pre-symptomatic detection and diagnosis than RGB-based approaches, because more subtle and more specific changes in reflectance or transmission within narrower spectral bands can be detected and signals outside of the visible spectral range can be exploited [51, 53–55]. Despite limitations of RGB-based approaches in this context, several interesting opportunities for studying details of pathogenesis may still be explored based on the proposed methodology. For example, distinct gene-for-gene interactions give rise to clearly visually distinguishable lesion phenotypes in tan spot of wheat caused by *Pyrenophora tritici-repentis* [57]. Similarly, we recently demonstrated the existence of a significant host-related genetic component in the degree of yellow halo formation around necrotic lesions of *Z. tritici* under natural epidemic development, which supports a similar hypothesis for this pathosystem [30]. More detailed analyses of contrasting but reproducible lesion phenotypes based on time-resolved imaging may reveal relationships between the occurrence of certain phenotypes and, for example, rates or duration of lesion expansion, or initial and final lesion size. In terms of early diagnosis, we note that, for example, the largest (central) lesion depicted in Fig. 1C was not diagnosed with confidence as STB by several experts in our lab until the state depicted in the top 4th panel (i.e. more than 48 h after signs of some unidentified leaf disorder became first apparent). We believe that to explore the principle feasibility of predicting future lesion properties in image time-series based on currently observed lesion phenotypes (see e.g [58]). represents an exciting frontier in the study of plant-pathogen interactions, which can be tackled with both RGB and hyperspectral imagery.

### Potential and limitations for practical application scenarios

Across all operators, leaf levels, cultivars, batches, and measurement conditions, on average approximately 100 leaves could be imaged in one working hour (including the search for the next marked plant in the next plot, identification of the relevant leaf on the marked stem, and an occasional basic quality check of captured images). We estimate that on average, the flattening of the leaf and the capturing of the image itself take less than 10 s. The proposed measurement setup is affordable, and the image acquisition protocol is standardized, therefore multiple devices could be operated in parallel by multiple operators. Our pilot data suggests that a measurement every two days is sufficient to capture the most relevant aspects of disease development, since lesions continue to grow for many days. Provided environmental conditions prevailing during the measurement intervals are properly taken into account (e.g., following the procedure proposed by Roth et al. [50]), samples could be allocated to separate batches to be measured on different days without introducing a bias.

Depending on the stability of the traits of interest and the relative importance of different potential sources of variance besides the genotype (such as e.g., leaf per se, leaf layer, leaf age, plant, plot, environmental conditions during the assessment period, year of assessment, etc.) more or less replicate measurements carried out over a longer or shorter period will have to be gathered to reliably estimate the effect of the genotype. These sources of variance remain to be accurately quantified, based on a larger data set and more detailed statistical analyses that take environmental conditions explicitly into account. Yet, even if many measurements must be made, a more accurate functional description of known sources of QR, as reported by Adhikari et al. [6] based on manual measurements, could be efficiently performed using the proposed methodology. Such knowledge can allow for more targeted crossings in breeding programs and can support the design of variety mixtures by maximizing complementarity of resistance mechanisms [5, 10, 59]. Combined with accurate time-integrated canopy-level measurements of plant health (e.g [15, 18, 19])., leaf level data and derived information on specific resistance components can aid the identification of components of resistance that are most closely associated with whole-plant resistance [5, 19]. Similarly, detailed information on disease development and its dependency on environmental conditions could be obtained for specific important cultivars to enable a fine-tuning of epidemiological models to the level of individual cultivars, for example in the context of variety testing. Better predictions of seasonal epidemics will improve management decisions in commercial farming.

The cost-benefit ratio of a high-resolution image in combination with the required processing tools is arguably considerably higher than that of, for example, a manual measurement of the length of the two major axes of a single lesion that should take at least as much time. However, the achieved throughput is, for the moment, likely insufficient to support regular screening in the context of large-scale genetic studies, even under a best-case scenario in terms of potential sources of variance to take into account. For example, for genome-wide association studies, several hundred plots would have to be monitored. While this is not impossible, the added value as compared to a more traditional and spatially coarse phenotyping approach using visual scoring (or digital approximations thereof [15, 18, 19]), will have to be carefully balanced against the additional costs incurred. The outcome of such considerations is likely to differ according to the pathosystem under study.

### Potential for further improvements of the method

Our approach offers significant gains in measurement throughput over traditional visual or manual assessments, especially if the number of symptoms and different properties assessed simultaneously is considered. A minor incremental improvement of the proposed method could be a better spatial arrangement of reference marks delimiting the ROI. Placing additional markers at both horizontal ends of the ROI will solve the issues in aligning the parts of the images representing the most distal and basal parts of the leaves. Image acquisition may be difficult to automate for further gains in throughput because of the need for physical intervention with specific leaves to ensure proper exposure of the leaf to the imaging device. However, an image registration process capable of aligning sequential images without the need for artificial marks could reduce the workload associated with our current method while at the same time increasing the analyzable leaf area present in each image. Though this may be very challenging for wheat leaves which do not have many distinct features on the leaf, nor a feature-rich contour that could guide a marker-free alignment [36, 37], novel deep-learning based approaches could offer significant potential for resolving this issue. At the level of image processing, a more explicit consideration of time differences between images during inference will allow refining leaf-level segmentation and detection results. Currently, inference is performed on each image of a time series in isolation. Finally, broadening the training database for the underlying symptom detection and segmentation models will further increase the precision and robustness of time-integrated phenotype extraction.

## Conclusions

We propose a methodology for rapid, non-destructive, and reproducible measurement of several key epidemiological parameters of Septoria tritici blotch using repeated RGB imagery. We demonstrated the capability of the proposed methods to accurately monitor the development of septoria tritici blotch and leaf rust diseases at the level of individual disease symptoms under field conditions. The derived image-based traits and their temporal evolution were generally in excellent agreement with corresponding visual impressions. Analysis of lesion size and lesion growth dynamics across cultivars indicated that lesion expansion under field conditions is under complex regulation. Yet, our method revealed genotype-specific progression of the number of infection events and of lesion expansion. These findings illustrate how repeated image-based disease monitoring can support decomposition and functional understanding of QR and help improve predictions of epidemiological development. The proposed methodology can be adapted to other pathosystems and can be extended to include additional aspects of disease that may better represent biological details of pathogenesis. While arguably still too time-consuming for routine use in the context of quantitative genetics, the proposed methods hold significant potential for detailed investigation of the spread of the disease within leaves and across leaf layers and will facilitate the identification of key host-specific and environmental drivers of seasonal epidemics.

## Electronic supplementary material

Below is the link to the electronic supplementary material.

Supplementary Material 1

## Data Availability

No datasets were generated or analysed during the current study.
