# Supplementary material for: SYMPATHIQUE: image-based tracking of symptoms and monitoring of pathogenesis to decompose quantitative disease resistance in the field
Source: Plant Methods. 2024 Nov 10;20:170. doi: 10.1186/s13007-024-01290-4 (PMC11552352; doi:10.1186/s13007-024-01290-4)
Supplement: Supplementary file 1 — Supplementary Material 1 [file 13007_2024_1290_MOESM1_ESM.pdf]

SYMPATHIQUE: Image-based tracking of Symptoms and monitoring of Pathogenesis to decompose Quantitative disease resistance in the field

Jonas Anderegg[1], Radek Zenkl[1], Norbert Kirchgessner[2], Andreas Hund[2], Achim Walter[2], Bruce A. McDonald[1]

[1]Plant Pathology Group, Institute of Integrative Biology, ETH Zurich, Zurich, Switzerland

[2]Crop Science Group, Institute of Agricultural Sciences, ETH Zurich, Zurich, Switzerland

*Corresponding author: Jonas Anderegg; E-mail: jonas.anderegg@usys.ethz.ch

Supplementary Materials

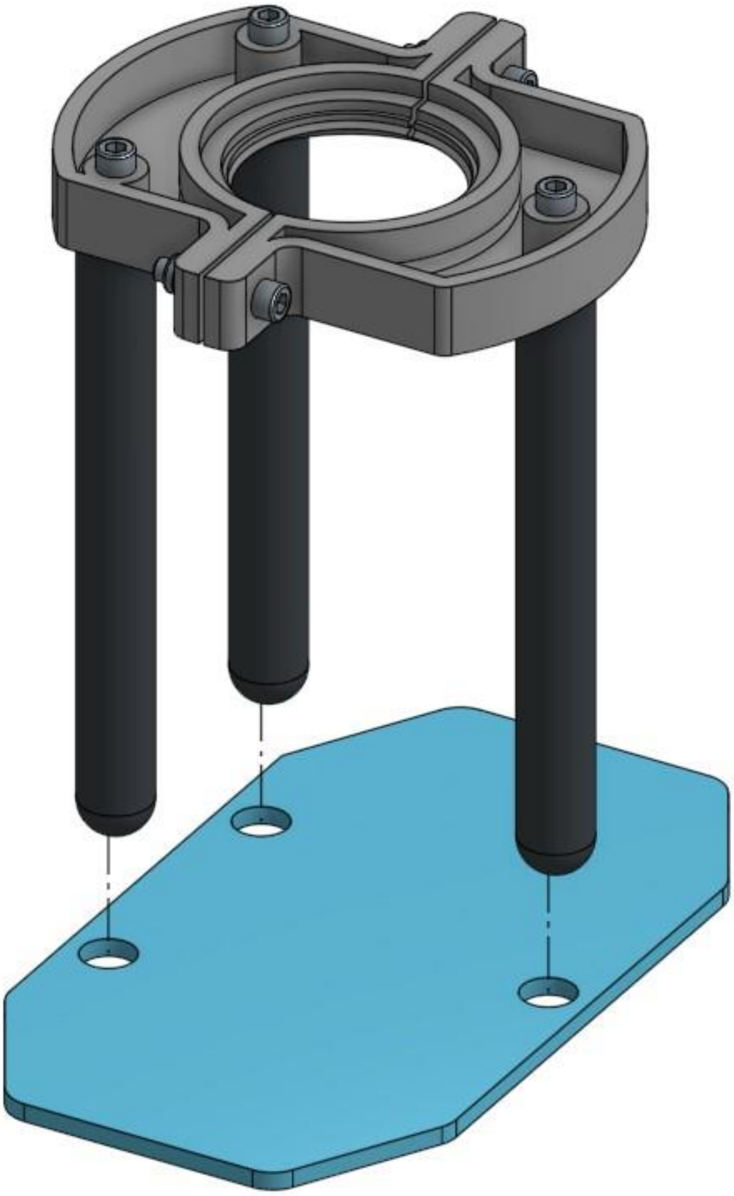

Supplementary Figure S 1 *Custom-developed imaging set-up for in-field acquisition of leaf image series. The set-up consisted of a 3D-printed spacer to be mounted directly onto the camera lens, and an acrylic glass base plate.*

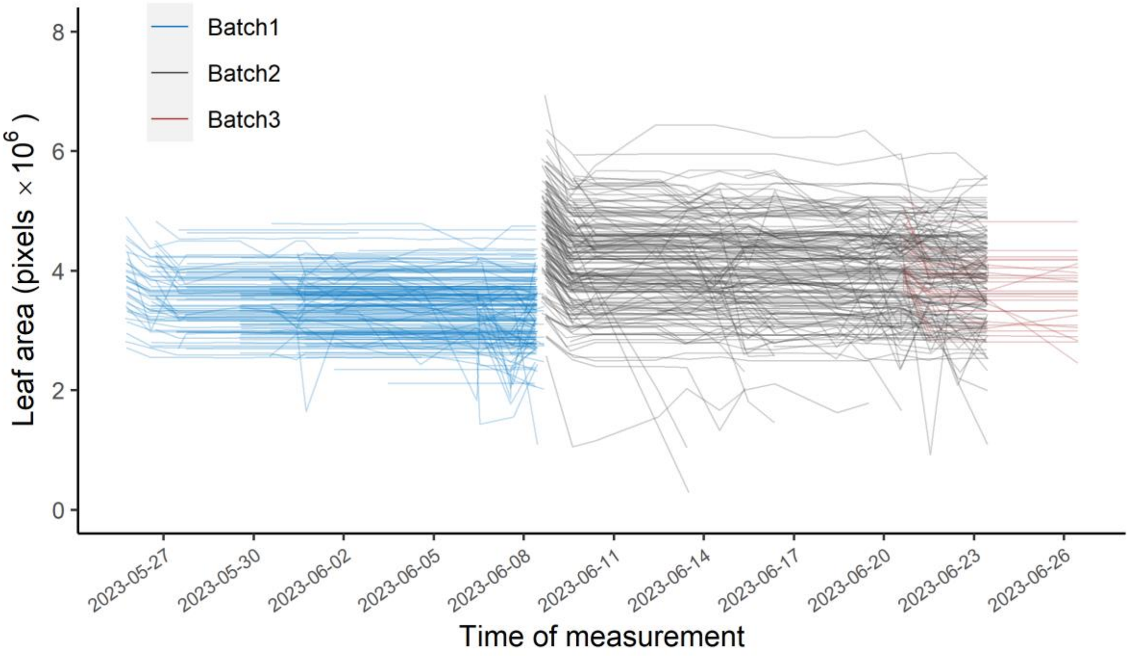

Supplementary Figure S 2 *Analyzable leaf area for all samples in the data set over time. Straight lines indicate samples for which the entire region of interest (ROI) could be registered to the ROI of the reference image (i.e., the first image of the series) in all images of a series. Deviations indicate partial failure of image alignment due to undetected marks or failure to correctly associate detected marks across images. Note that the initial decrease in all samples is because only the convex hull defined by the reference marks is transformed, but not the entire leaf area visible in the reference image. Data for a particular leaf was only exported upon appearance of first lesions on that leaf. Batch 1 corresponds to penultimate leaves, whereas batches 2 and 3 correspond to flag leaves.*

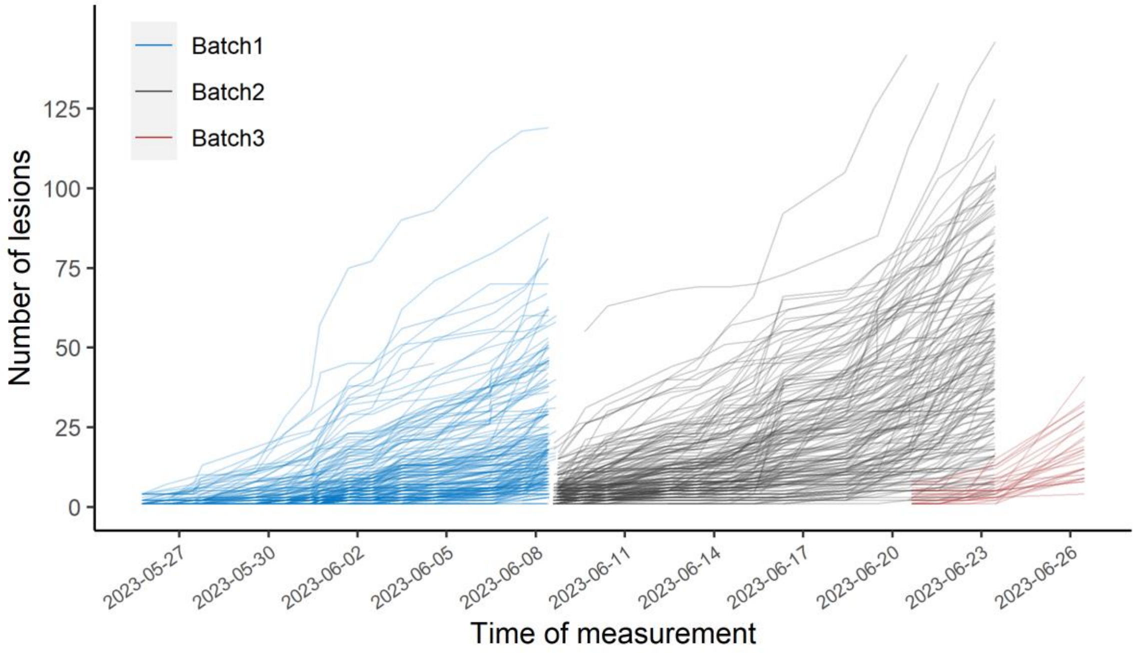

Supplementary Figure S 3 *Number of lesions on all samples in the data set over time. Each line represents one leaf measured multiple times over the depicted period. Data for a particular leaf was only exported upon appearance of first lesions on that leaf (leaves that stayed asymptomatic throughout the measurement period are not shown). Batch 1 mostly corresponds to penultimate leaves, whereas batches 2 and 3 correspond to flag leaves.*

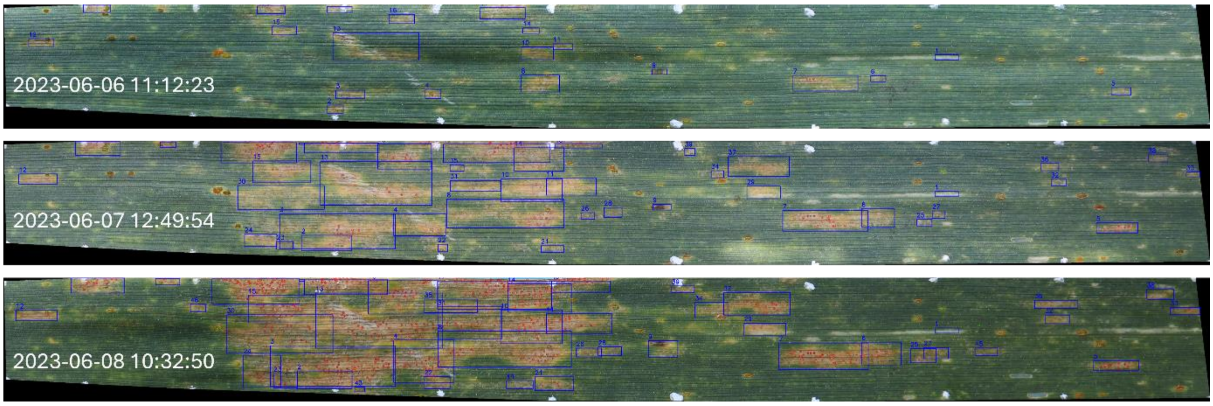

***Supplementary Figure S 4** Registered images for an example leaf with multiple rapidly expanding lesions and a very high pycnidia emergence rate. Blue bounding boxes denote individual detected lesions and are numbered according to the order of lesion appearance. Green circles mark detected rust pustules, and red circles mark detected pycnidia. Indicated datetimes refer to the timepoint of image acquisition. These images highlight some remaining issues with the underlying detection and segmentation models (for example, lesion 1 is most likely not a necrotic lesion but rather represents some physical damage). Note that a high measurement frequency is required to identify initially separate lesions before they coalesce to larger blotches in this case.*

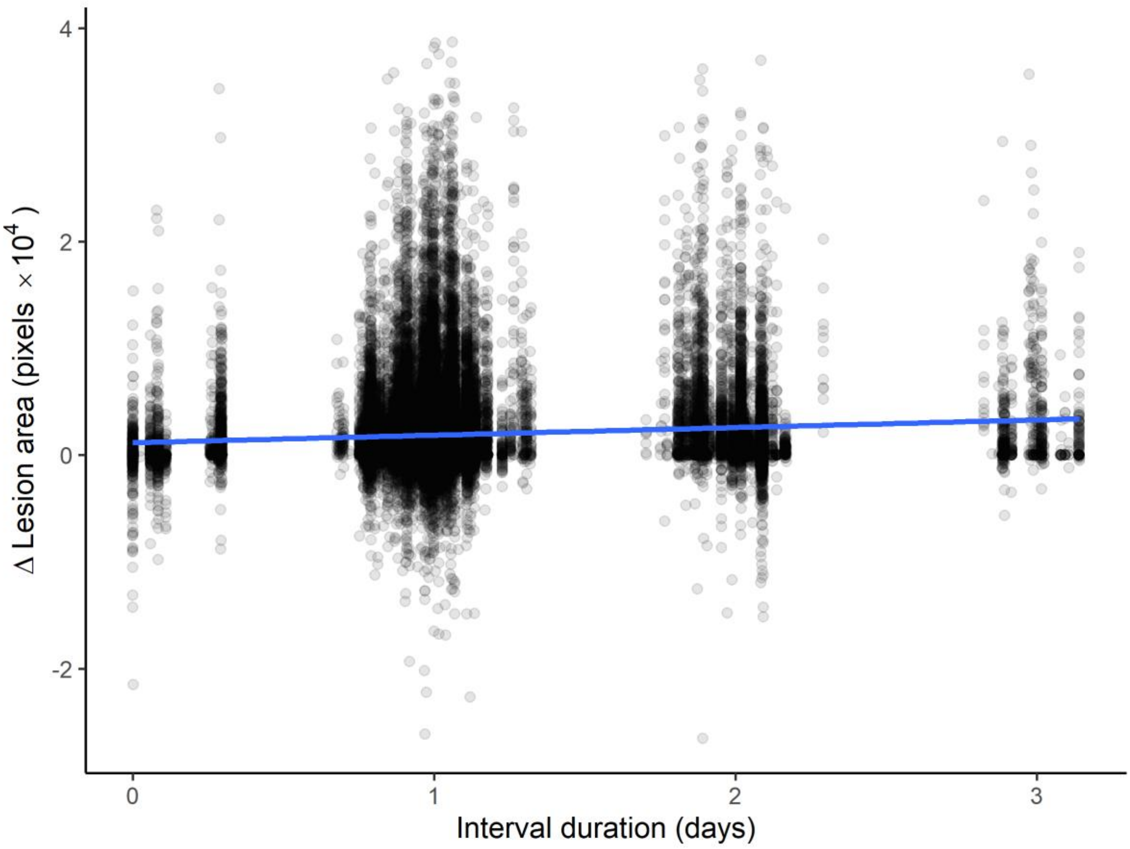

Supplementary Figure S 5 *Changes in lesion area as a function of the duration of the measurement interval. The blue line represents the least squares line.*

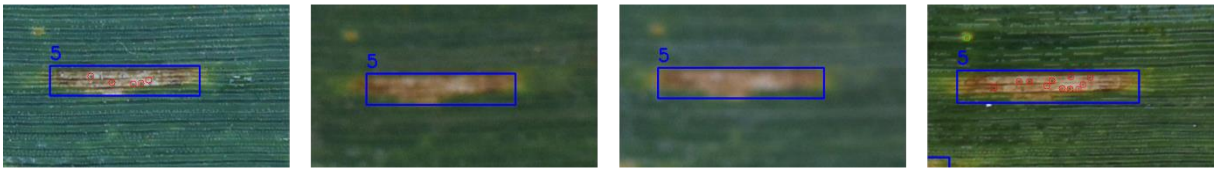

Supplementary Figure S 6 *Deviations from expected regular temporal patterns in the number of pycnidia per lesion are often related to occasional image quality issues. Here, an operator error resulted in certain images of a time series being out of focus, which affected pycnidia detection (red circles). Images are from four consecutive measurements and show a lesion on a leaf of cultivar 'CH Claro'. The period of interest is highlighted in Figure 7. The green circle represents a predicted rust pustule.*

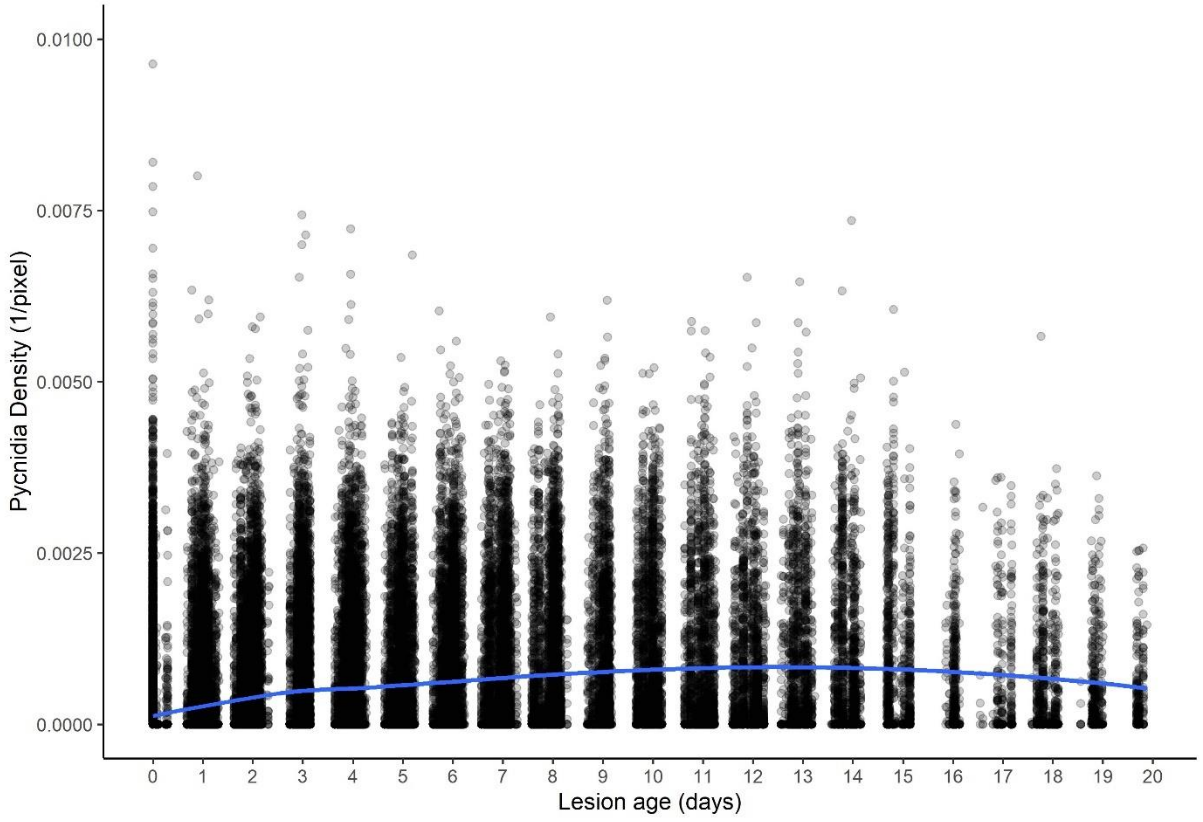

Supplementary Figure S 7 *Pycnidia density (number of pycnidia per total leaf area in pixels) as a function of lesion age. A Loess fit line was added to the scatter plot to show the trend of the relationship.*

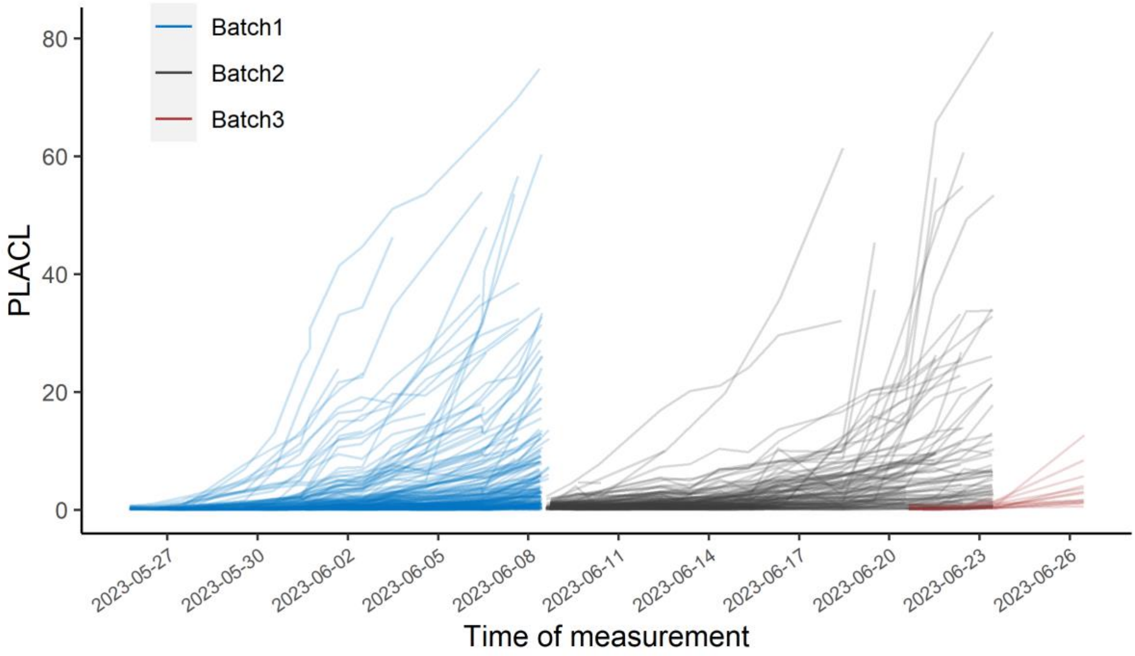

Supplementary Figure S 8 *Percentage leaf area covered by lesions (PLACL) on all samples in the data set over time. Each line represents one leaf measured multiple times over the depicted period. Note that measurements were retained only if the analyzable leaf area was at least 90% of the initial leaf area. This was necessary because PLACL is only comparable if calculated for a constant region of interest. Data for a particular leaf was only exported upon appearance of first lesions on that leaf (leaves that stayed asymptomatic throughout the measurement period are not shown). Batch 1 mostly corresponds to penultimate leaves, whereas batches 2 and 3 correspond to flag leaves.*

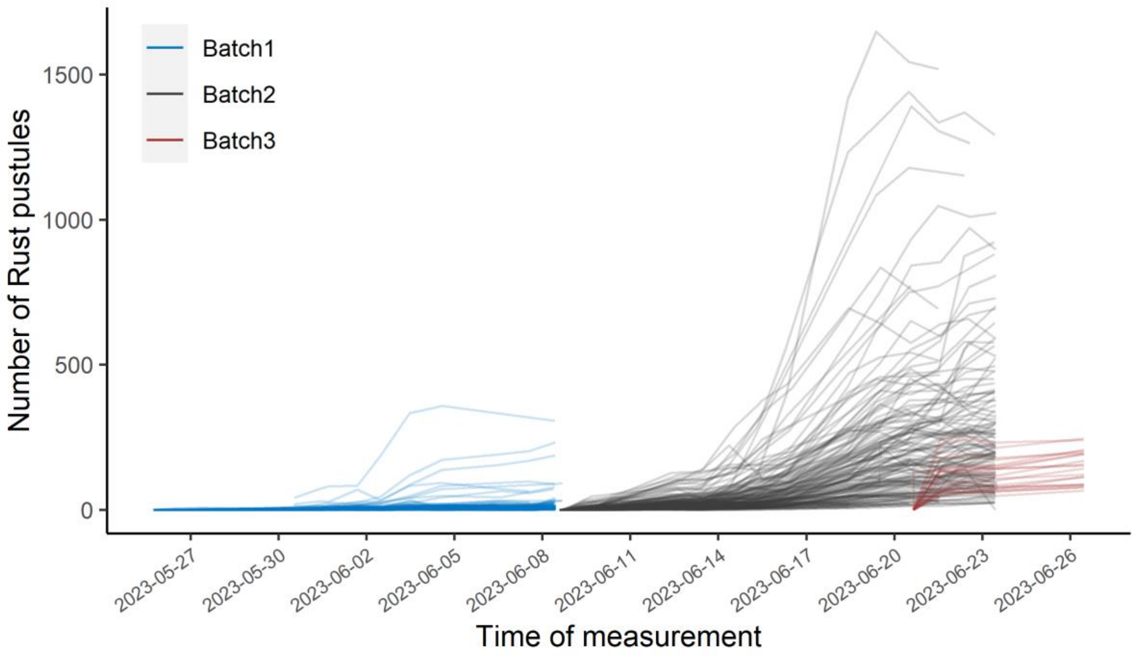

Supplementary Figure S 9 *Number of rust pustules detected on all samples in the data set over time. Each line represents one leaf measured multiple times over the depicted period. Note that measurements were retained only if the analyzable leaf area was at least 90% of the initial leaf area. This was necessary because the number of rust pustules detected is only comparable if calculated for a constant region of interest. Data for a particular leaf was only exported upon appearance of first necrotic lesions on that leaf (leaves that stayed asymptomatic throughout the measurement period are not shown). Batch 1 mostly corresponds to penultimate leaves, whereas batches 2 and 3 correspond to flag leaves.*

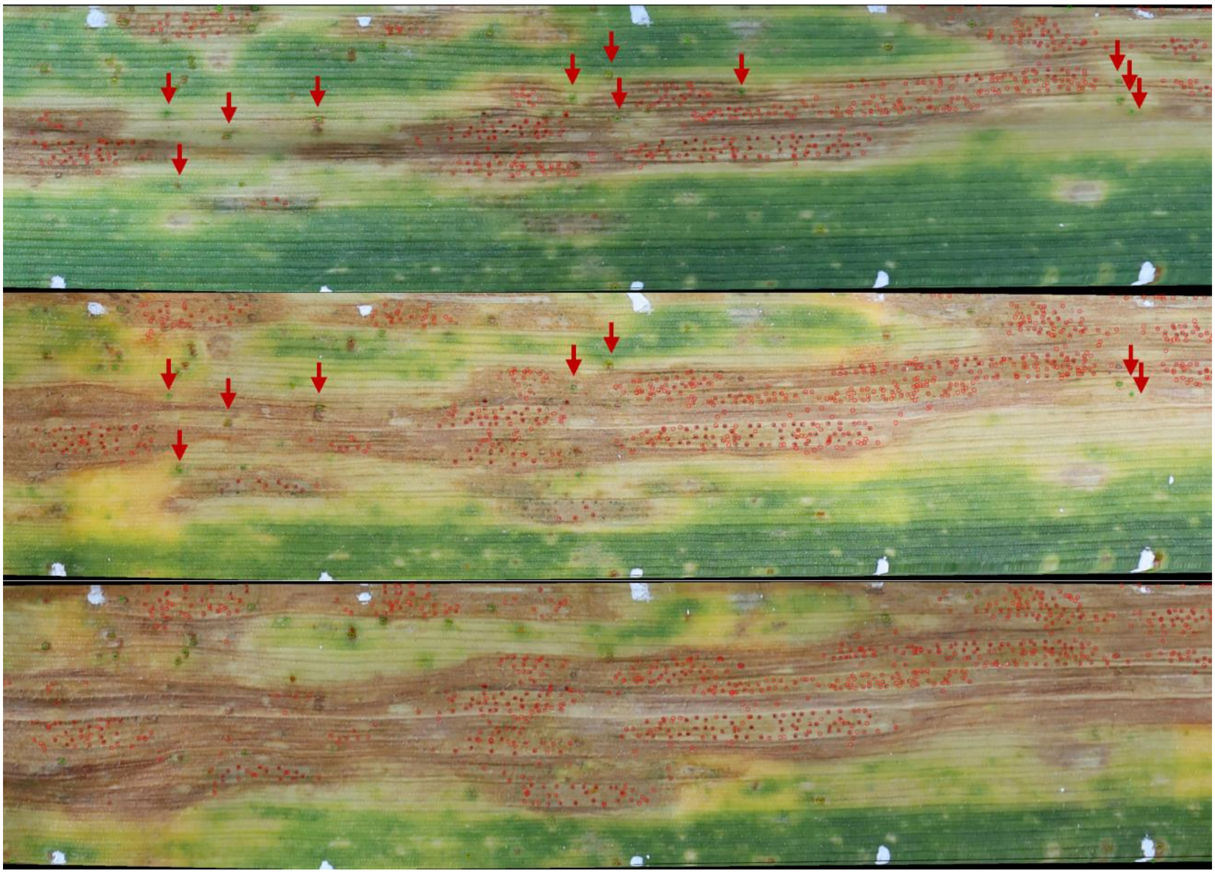

Supplementary Figure S 10 *Cutout from registered images of a leaf at advanced stages of disease development and with the beginning of physiological senescence. Detection of rust pustules in severely diseased or old leaves is challenging and can explain the decrease observed occasionally in the total number of detected rust pustules per leaf towards the end of the measurement period. Red arrows mark rust pustules that were initially detected but were not detected in images of the same leaf at later stages. Green and red circles mark detected rust pustules and pycnidia, respectively. Note that the segmentations have been omitted for a better visibility of the detections.*
